# The adaptation of health care marketing to the digital era

**Published:** 2017

**Authors:** G Radu, M Solomon, CM Gheorghe, M Hostiuc, IA Bulescu, VL Purcarea

**Affiliations:** * Radiology and Medical Imaging, University of Medicine and Pharmacy, Craiova, Romania

**Keywords:** healthcare, marketing, digital, Internet, promoting

## Abstract

The purpose of health care marketing is to learn and understand the needs and desires of prospective patients in order to be able to meet those necessities at the highest standards. A big advantage is the targeting capability of the electronic media that has led to its being used by managers of marketing in medical institutions as means of advertisement when they develop the marketing strategies. Regarding social media, it is safe to say that there are communication platforms that can promote certain behaviours thus influencing decision-making. Through social media, people stay in touch with other people and they can provide a mean for medical institutions to permanently communicate with the existing patients or with the potential ones. In addition, social media can be used in advertising and promoting strategies, by posting information about discounts, offers and advantages of accessing the products provided by a certain institution. A study was conducted on 126 patients of a dental clinic in Bucharest.

126 new patients were selected on a period of 22 months from January 2015 until October 2016. The patients never had any treatment in this clinic and were influenced by the Internet to seek for dental care services. The purpose of this study was to evaluate the digital methods of promoting medical services, bringing new patients to a clinic.

The results of the study demonstrated the need for digital methods of promoting medical care services in order to expand a business. A strategic way of thinking in this case implied attracting new patients and offering them quality health care services, which ensured their satisfaction and the probability of their recommending the health facility further. This study revealed an important role of social networking sites in promoting. This high response was probably responsible due to targeted promoting services. Almost all the new patients who completed the form will remain patients of this clinic in future.

Confronted with the exponential growth in the use of electronics by consumers, marketing experts have come up with new ways and means of incorporating the new technologies in their strategies. 

The purpose of health care marketing is to learn and understand the needs and desires of prospective patients in order to be able to meet those necessities at the highest standards.

The Internet, e-mail and social media are not only cheaper methods than the direct marketing, but they also create the opportunity to market a service to virtual costumers, breaking the barrier of distance and making consumers aware of the service being offered at any time and at any place. 

Another important advantage is the targeting capability of the electronic media that has led to its being used by managers of marketing in medical institutions as means of advertisement when they develop the marketing strategies [**1**].

Investing in a good website is always a good idea. An interactive and user-friendly website that offers lots of information represents a great advertising platform. It can be a source of information for potential patients, employees or other staff members. The patients can analyse a description of the medical facility, services being provided, as well as read other customers’ reviews, browse photos or compare prices and offers with the ones of other clinics. An advantage is that any person using a search engine can access the website with the help of keywords. This way, potential patients are being targeted. The medical organization can communicate regarding the institution itself or its product offerings through the website, permitting the Internet user to search exactly the information wanted, as well as maintain a dialogue between the consumers and the clinic [**2**].

Marketing-wise, a website will be successful only if it is integrated in the marketing strategies. First, the patient should be encouraged to access the website by the employees, by flyers or by banners as well as by other commercials in the media. Also, the marketing managers can elaborate offers, promotions and discounts that can be accessed through the website and that will encourage potential clients to visit the medical establishment [**3**].

The drawbacks of incorporating websites in the marketing strategies of a company should also be taken into consideration. First, the advantage of being a cheaper advertising method meets the disadvantage of having to invest in other forms of online and offline media in order to promote the website; otherwise, it will not be successful. Thus, the additional expenses needed to attract Internet users towards it can be considerable. Next, the need to keep the website up-to-date should also be taken into account. Out-dated information may have a negative impact on the institution’s image. The design of the homepage is of great importance in drawing the patients in and encouraging them to further search the site. Finally yet importantly, the fact that any Internet user can access the website brings the threat of competition searching the website and using information to its own advantage. Caution is recommended when deciding what type of information will be posted on an organization’s website [**4**].

Other types of Internet tools that can be integrated in the marketing strategies of a medical organization are links, pop-ups, and banner advertisements on other websites or on social media platforms, which lead the potential visitors to the clinic’s own website [**5**].

Regarding social media, it is safe to say that there are communication platforms that can promote certain behaviours, influencing decision-making. Through social media, people stay in touch with other people and they can provide a mean for medical institutions to permanently communicate with the existing patients or with the potential ones. In addition, social media can be used in advertising and promoting strategies, by posting information about discounts, offers and advantages of accessing the products provided by a certain institution [**6**].

Contacting the existing patients through e-mail in order to inform them about the special offers and the new available services is another great use of modern technology in marketing. Nevertheless, it has been determined that, in order for it to be a successful strategy, the recipient targeted by the e-mail should already be aware of the medical institution. Otherwise, the e-mail might quickly be sent to the recycle bin, without it even being read. The e-mail headline should also be attention catching in order to determine the recipient to open the mail. The advantage of the e-mail over direct postal mail is the minimal expense needed, and, the information will reach its destination immediately. A downside regarding the use of e-mails in marketing campaigns is that, although pictures can be sent via e-mail, promotional gifts cannot. Also, the absence of face-to-face contact limits the ability to persuade a potential patient to try-out the services offered by the medical institution [**7**].

Another way of incorporating modern technology in marketing is the use of mobile or cell phones. Considering that this is a device that every patient has at his side at any time, it is a certain method for the medical organization to keep in contact with its clients. Whether it is through text messages, voicemail, phone calls or Internet, the patients can receive information about new items the clinic has to offer, prices, discounts, validate appointments or get information. It has been established that, in comparison with e-mail, a text message is more likely to be read the moment it is received [**8**].

Finally yet importantly, the impact of digital television and radio on marketing should also be considered. In comparison with the analogue systems, digital television and radio offers a better interaction opportunity, a better transmission quality and more choices in terms of programs and channels. Combining the interactivity of the Internet with the visual impact of television, this type of technology is more effective for marketing communications. Also, the variety of channels allows a better profiling and targeting [**9**].

A study on 126 patients of a dental clinic in Bucharest was conducted. 

126 new patients were selected on a period of 22 months, from January 2015 until October 2016. The patients never had any treatment in this clinic and were influenced by the Internet to seek for dental care services. 

The purpose of this study was to evaluate the digital methods of promoting medical services, bringing new patients to a clinic.

All this information was required to fill in a questionnaire

1. Please fill in the following: sex…. Age…. 

2. Have you ever been a patient of this clinic? Yes/ No (if no, please continue to next question))

3. Were you influenced by any method of promoting on the Internet of this dental practice to seek medical care in this unit? Yes/ No (if yes, please continue to next question)

4. Which of the following methods influenced you to seek care in this unit? Facebook/ Website (Google sponsor)/ discount voucher or coupons websites/ e-mail 

5. Did you use to follow this clinic’s Facebook page before becoming a patient, or any other social networking sites?

6. Are you going to follow our Facebook page?

7. Are you going to remain a patient of this clinic?

8. Would you like to receive information about our offers and promotions through e-mail?

Based on the answers offered to the second questions, all the patients who have already been patients of this clinic were eliminated from the start. In 22 months, 126 patients were admitted in this study. 

Their average age was 30.2 years old, the youngest patient was 6 years old and was brought by his parents for a baby tooth extraction. The parents knew about this clinic from a social networking website. The oldest was 42 years old. 75 of these patients were women and 51 men.

**Fig. 1:**
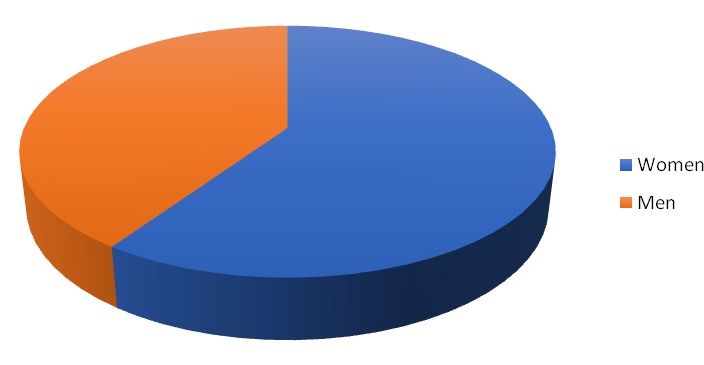
Women vs. men chart

The answers to the third question showed an important role of promotions on the Internet regarding the influencing of potential future patients in seeking care in a precise medical unit. 78.57% of them, representing 99 patients, were influenced by Internet in choosing the medical unit. This answer demonstrated an important role played by digital methods in promoting a certain service. 

For the forth question on the list, the patients had to note down which of the digital methods influenced them in choosing this medical practice. It is known that the social networking website, such as Facebook, allows the promotion of your business to *targeted* people with a cost (targeted by: sex, age, locations, interest). We consider this method efficient. The same thing can be done with the search engines such as Google sponsor. At this question, the majority was determined to choose this medical clinic because of promotions on different social networking sites. The difference between these methods was probably because of the targeted possibility of promotion to some certain people.

**Fig. 2:**
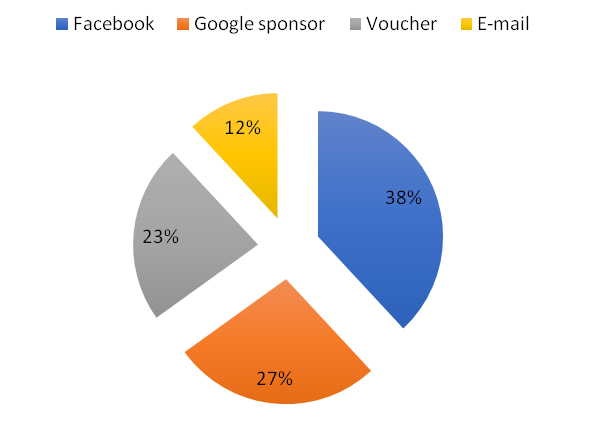
Digital methods of promoting services

The answers to the fifth question revealed the importance of activity on social networking sites. Almost 85% of the patients were following at least one page of the social networking sites. The importance of social networking sites activity of a medical practice is revealed by the answer to that question.

50% of the patients who do not follow the social networking sites said that they would probably follow the medical practice social networking sites in future. 90% of the patients believed that they would come again at this medical practice.

The results of the study demonstrated the need for digital methods of promoting medical care services in order to expand a business. A strategic way of thinking in this case implies attracting new patients and offering them quality health care services, which ensures their satisfaction and the probability for them to recommend the health facility further. This study revealed an important role of social networking sites in promoting. This high response was probably responsible due to the targeted promoting services. Almost all the new patients who completed the form will remain patients of this clinic in future.

All authors have equally contributed to this article.
